# Alternative treatments in advanced hepatocellular carcinoma patients with progressive disease after sorafenib treatment: a prospective multicenter cohort study

**DOI:** 10.18632/oncotarget.10794

**Published:** 2016-07-23

**Authors:** Masahito Nakano, Masatoshi Tanaka, Ryoko Kuromatsu, Hiroaki Nagamatsu, Manabu Satani, Takashi Niizeki, Shusuke Okamura, Hideki Iwamoto, Shigeo Shimose, Tomotake Shirono, Yu Noda, Hironori Koga, Takuji Torimura, Kurume Liver Cancer Study Group of Japan

**Affiliations:** ^1^ Division of Gastroenterology, Department of Medicine, Kurume University School of Medicine, Kurume, Fukuoka, Japan; ^2^ Yokokura Hospital, Miyama, Fukuoka, Japan; ^3^ Yame General Hospital, Yame, Fukuoka, Japan

**Keywords:** sorafenib, hepatocellular carcinoma, progressive disease, follow-up treatments

## Abstract

Sorafenib is an oral multikinase inhibitor that has been approved to treat advanced hepatocellular carcinoma (HCC), though it is unclear how much benefit advanced HCC patients with progressive disease (PD) derive from sorafenib treatment. This study aimed to assess survival risk factors and evaluate therapeutic strategies for advanced HCC patients with PD after sorafenib treatment. We analyzed the clinical data and treatment outcomes for 315 consecutive advanced HCC patients treated with sorafenib. Univariate analyses of overall survival identified therapeutic effect as an independent risk factor in all patients. Among all patients, 141 developed PD. Of those, 58 (41%) were treated with sorafenib monotherapy, 70 (50%) with agents other than sorafenib, and 13 (9%) were not treated at all. The median survival time was 6.1 months for PD patients with sorafenib monotherapy and 12.2 months for those administered alternative treatments (p < 0.0001). Our results indicated that sorafenib treatment may have negative long-term therapeutic effects in advanced HCC patients with PD, and that alternative treatments should be considered for these patients after sorafenib administration.

## INTRODUCTION

Hepatocellular carcinoma (HCC), one of the most prevalent types of cancer worldwide [1–4], is the major histological subtype of liver cancer, accounting for about four fifths of total primary liver cancer cases [5, 6]. The therapy landscape for late-stage HCC has changed with the advent of molecular-targeted therapy [7]. Sorafenib, a relatively recent molecular therapy for late-stage HCC, has been used in Japan since mid 2009 [4, 8–10].

The tyrosine kinase inhibitor sorafenib has been demonstrated to induce tumor cell apoptosis. Its targets include multiple kinases such as vascular-endothelial and platelet-derived growth factor receptors, as well as the proto-oncoprotein c-Raf and other molecules [11–14]. Administration of sorafenib to advanced HCC patients was demonstrated to be efficacious and safe in the Sorafenib HCC Assessment Randomized Protocol (SHARP) [15] and Asia-Pacific studies [16]; however, the survival benefits of the drug are limited even when combined with immunomodulatory agents [14, 17]. As the duration of survival benefit is determined by disease condition, patients undergo sorafenib treatment with the aim of maintaining stable disease (SD). In spite of the multiple clinical trials conducted to test molecular therapeutic agents other than sorafenib, no agent has been found to have an efficacy superior to that of sorafenib to treat unresectable HCC [18–21].

Second-line therapies for late-stage HCC after sorafenib are lacking, and the selection of a treatment modality after first progression remains controversial [14]. For patients who develop progressive disease (PD) after being treated with sorafenib, the medication is often discontinued in favor of second-line trials [22, 23]. Consequently, continued sorafenib treatment has been selective for patients with late-stage HCC who present tumor progression; however, the efficacy of sorafenib for advanced HCC patients with continued treatment has not been determined [4, 14].

Here, we aimed to determine whether continued sorafenib treatment in advanced HCC patients who develop PD is beneficial. We identified prognostic indicators in patients with late-stage HCC treated with sorafenib, and evaluated alternative treatments in those patients who develop PD after sorafenib.

## MATERIALS AND METHODS

### Patients

Eligibility criteria for this study were similar to those of the SHARP trial [15], and were equivalent to the criteria we employed in our previous studies [4, 9]. Briefly, all enrolled patients met the following requirements: (a) Eastern Cooperative Oncology Group performance status of 0–1, (b) measurable disease using the Response Evaluation Criteria in Solid Tumors (RECIST) [24], (c) Child-Pugh class A or B liver function, (d) leukocyte count of ≥2,000/mm^3^, (e) platelet count of ≥50×10^9^/L, (f) hemoglobin level of ≥8.5 g/dL, (g) serum creatinine level of <1.5 mg/dL, and (h) no ascites or encephalopathy. We enrolled 315 consecutive patients who were diagnosed with advanced HCC between May 2009 and September 2014 and who received sorafenib in this study. HCC was either confirmed histologically or diagnosed using noninvasive criteria according to the European Association for the Study of Liver [25]. Enrolled patients were treated with sorafenib at one of the 14 experienced member institutions of the Kurume Liver Cancer Study Group of Japan: Asakura Medical Association Hospital, Chikugo City Hospital, Kurume General Hospital, Kurume University Medical Center, Kurume University School of Medicine, Kyushu Medical Center, Nagata Hospital, Ōmuta City Hospital, Saga Central Hospital, Social Insurance Tagawa Hospital, St. Mary's Hospital, Tobata Kyoritsu Hospital, Yame General Hospital, and Yokokura Hospital. The primary outcome of this study was overall survival time, which was defined as the time from initiation of sorafenib treatment to the date of death or the patient's last follow-up. Relevant data from all patients’ clinical records, including medical history, laboratory results, radiological findings, histological results, and survival data, as well as the dosage and adverse events associated with sorafenib therapy, were prospectively collected. The study protocol was approved by the Ethics Committee of Kurume University (No. 10009) and the University Hospital Medical Information Network (UMIN) Center (No. UMIN000007427), and conformed to the guidelines of the 1975 Declaration of Helsinki. Patients were given comprehensive information on the details of the clinical study, and each provided written informed consent prior to participation.

### Diagnosis

Intrahepatic lesions and vascular invasion were diagnosed using a combination of contrast-enhanced computed tomography (CT), magnetic resonance imaging (MRI), ultrasonography (US), and digital subtraction angiography. Additionally, alpha-fetoprotein (AFP), lens culinaris agglutinin-reactive fraction of AFP (AFP-L3), and des-gamma-carboxy prothrombin (DCP) serum levels were measured up to one month before treatment. Intra-abdominal metastases were detected via abdominal CT, MRI, and US, which were performed to evaluate intrahepatic lesions. Pulmonary lesions were detected on chest radiography or CT, which was routinely performed up to one month before treatment. Additional examinations, such as bone scintigraphy and brain CT or MRI, were indicated when symptoms attributable to extrahepatic metastasis appeared. These examinations were also conducted when AFP, AFP-L3, or DCP levels were elevated in a manner that could not be explained by the status of the intrahepatic lesions [25]. Tumor stage was determined according to the Barcelona Clinic Liver Cancer (BCLC) staging classification [26].

### Sorafenib treatment

Performance status was used to determine the initial sorafenib dose, at the discretion of the chief physician. Discontinuation and dose reduction were allowed based on tolerance. Side effects of sorafenib treatment were documented according to the National Cancer Institute's Common Terminology Criteria for Adverse Events (CTCAE), version 4.0. Treatments were discontinued upon development of CTCAE grade 3 or higher adverse events with the exception of a platelet count of <25×10^9^/L and a leukocyte count of <1,500/mm^3^.

### Assessment of tumor response

Imaging studies were performed four weeks after the initiation of sorafenib treatment and every 4–6 weeks thereafter to assess tumor response. The assessment was conducted according to the RECIST, version 1.1 [24] as follows: complete response (CR), all measurable lesions disappeared for more than four weeks; partial response (PR), the sum of the diameters of the largest target lesions decreased by more than 30%, and no new lesions developed for more than four weeks; PD, the sum of the largest diameters increased by more than 20%, or a new lesion appeared; and SD, where neither PR nor PD was observed [27]. Patients who died before their first radiographic assessment were classified as having PD. The time to radiologic progression was defined as the time from sorafenib treatment initiation to disease progression. Data from patients who died without tumor progression were censored. The disease-control rate was defined, on the basis of independent radiologic review, as the percentage of patients whose best-response RECIST rating of CR, PR, or SD was maintained for at least one month after the first demonstration of such a rating.

### Statistical analysis

Baseline patient characteristics were analyzed using descriptive statistical methods. Survival curves were calculated using the Kaplan-Meier method. Univariate analysis of survival curves was performed using the log-rank test. A *p*-value of <0.05 was considered statistically significant. The Cox proportional-hazards model was used to evaluate the interaction between baseline characteristics and the effect of sorafenib on overall survival. The JMP software (SAS Institute, Inc., Cary, NC, USA), version 11, was used for all analyses.

## RESULTS

### Patient characteristics

Some of the results in our present study are consistent with those reported in a previous study by our group [4]. Here, we also investigated the benefits of alternative treatments in patients with late-stage HCC who presented PD after being treated with sorafenib. The population of patients was composed of 246 (78%) men and 69 (22%) women. The mean age of patients was 72 years (Table 1). The main causes of HCC were chronic hepatitis C (n = 195; 62%) and hepatitis B (n = 57; 18%) virus infections. Of the patients enrolled in our study, 161 (51%) had a Child-Pugh score of 5, and 104 (33%) had a Child-Pugh score of 6 while 265 (84%) patients had Child-Pugh class A and 50 (16%) had class B liver cirrhosis. Per the BCLC staging system, 101 (32%) patients were in stage B HCC, and 214 (68%) were in stage C. Prior to sorafenib administration, 280 (89%) patients had undergone surgical, loco-regional, or pharmacologic treatment. Of these, 178 were treated with transcatheter arterial chemoembolization (TACE), 109 were underwent hepatic arterial infusion chemotherapy (HAIC), 93 underwent hepatic resection, and 77 had radiofrequency ablation.

**Table 1:** Characteristics of the total cohort (no. with % and median with range)

Variable	
Age - median in years [range]	72 [33 - 94]
Gender - n (%)	
Male	246 (78)
Female	69 (22)
Etiology - n (%)	
HBV	57 (18)
HCV	195 (62)
both negative	63 (20)
Child-Pugh class - n (%)	
A	265 (84)
B	50 (16)
Tumor stage - n (%)	
BCLC-B	101 (32)
BCLC-C	214 (68)
Initial sorafenib dose - n (%)	
400mg	217 (69)
800mg	98 (31)
Extrahepatic metastasis- n (%)	178 (57)
Lung	105 (34)
Bone	40 (13)
Lymph node	38 (12)
Peritoneum	17 (5)
Adrenal gland	11 (3)
Macrovascular invasion- n (%)	
Presence	82 (26)
Absence	233 (74)
Albumin - median in g / L [range]	3.50 [2.39 - 4.70]
Total bilirubin - median in mg / dL [range]	0.78 [0.15 - 3.70]
Prothrombin time - median in % [range]	83.3 [10.8 - 136.0]
AFP - median in ng / mL [range]	100 [1 - 987600]
AFP L3- median in % [range]	22.3 [0.0 – 99.6]
DCP - median in mAU / mL [range]	738 [2 - 621000]

HBV = hepatitis B virus; HCV = hepatitis C virus; BCLC= Barcelona Clinic Liver Cancer; AFP = Alpha-fetoprotein; DCP = Des-gamma-carboxy prothrombin

### Treatment compliance

The daily dose of oral sorafenib was 400 mg for 217 patients and 800 mg for 98 patients. By the end of the follow-up period, 277 patients had discontinued treatment for a variety of reasons including adverse reactions in 150 patients, radiologic and symptomatic progression in 92, and worsening of performance status in 22; moreover, 13 patients requested cessation of their treatment.

### Overall response and efficacy

Of the enrolled patients, 261 (83%) underwent sorafenib treatment for longer than a month. The median duration of sorafenib treatment was 3.6 months (range: 0.1–53.5 months), and the median follow-up period was 8.7 months (range: 0.4–57.3 months). Fifty-four patients who received sorafenib for less than a month were treated with other therapeutic modalities, including TACE, HAIC, systemic chemotherapy, or radiofrequency ablation. A total of 230 patients (73%) died during the observation period while 85 (27%) survived through the follow-up period. Table 2 shows the results at the first radiologic assessment according to the RECIST; the rate of disease control was 49%.

**Table 2:** Therapeutic effects in all patients (n =315)

Therapeutic effect	n (%)
PR	19 (6)
SD	136 (43)
PD	141 (45)
Not evaluable	19 (6)

PR = partial response; SD = stable disease; PD = progressive disease

### Factors correlated with survival outcome

**Figure 1:**
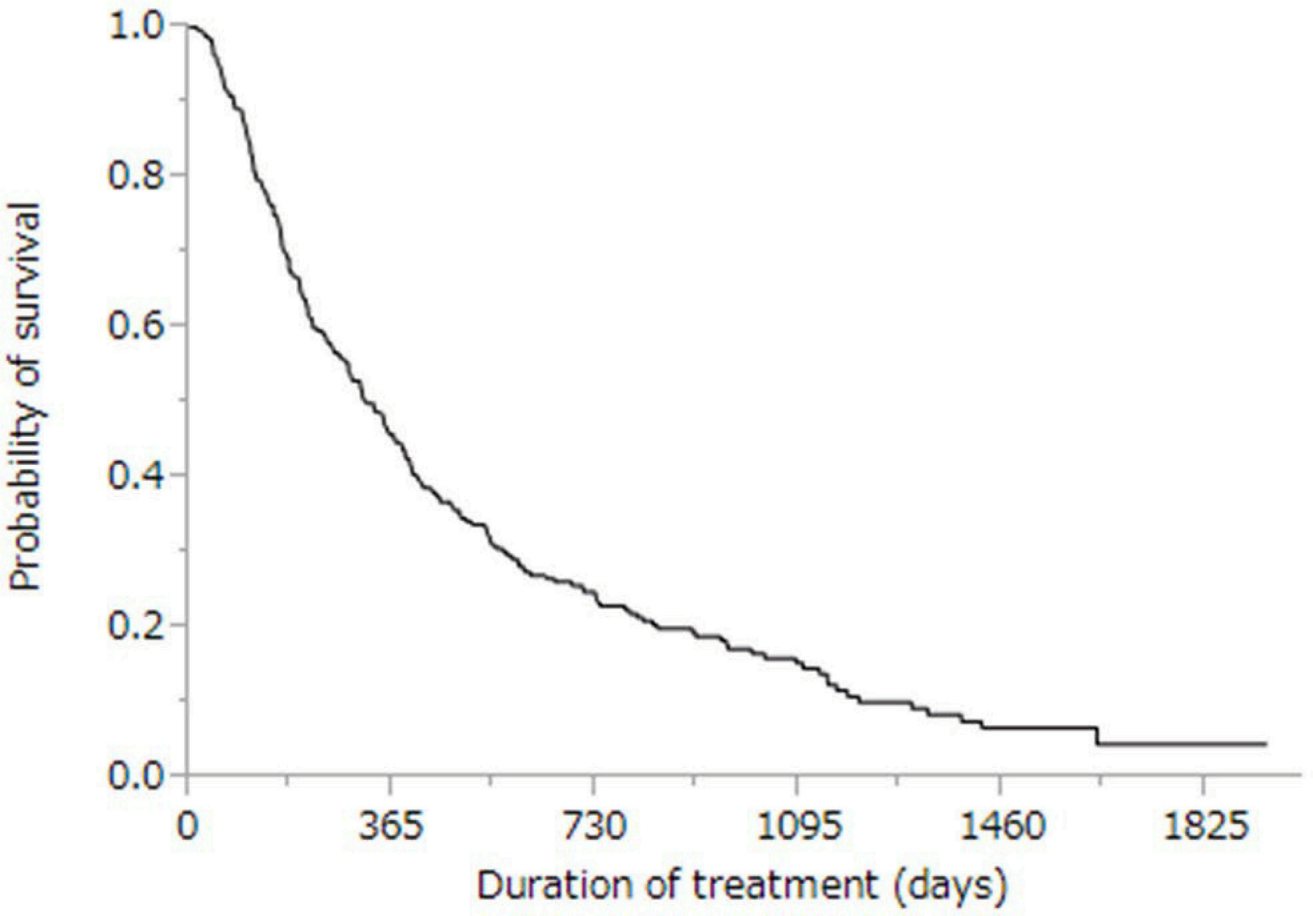
Kaplan-Meier analysis of overall survival of enrolled patients Median survival time was 10.6 months, and the one-year survival rate was 46%.

**Figure 2:**
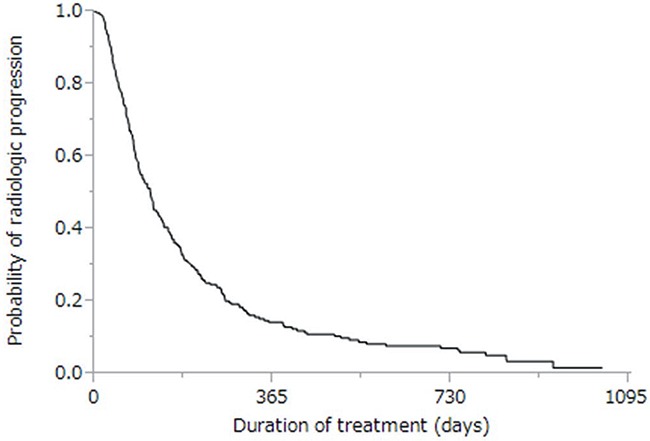
Kaplan-Meier analysis of radiologic progression-free survival of enrolled patients Median survival time was 3.9 months.

**Figure 3:**
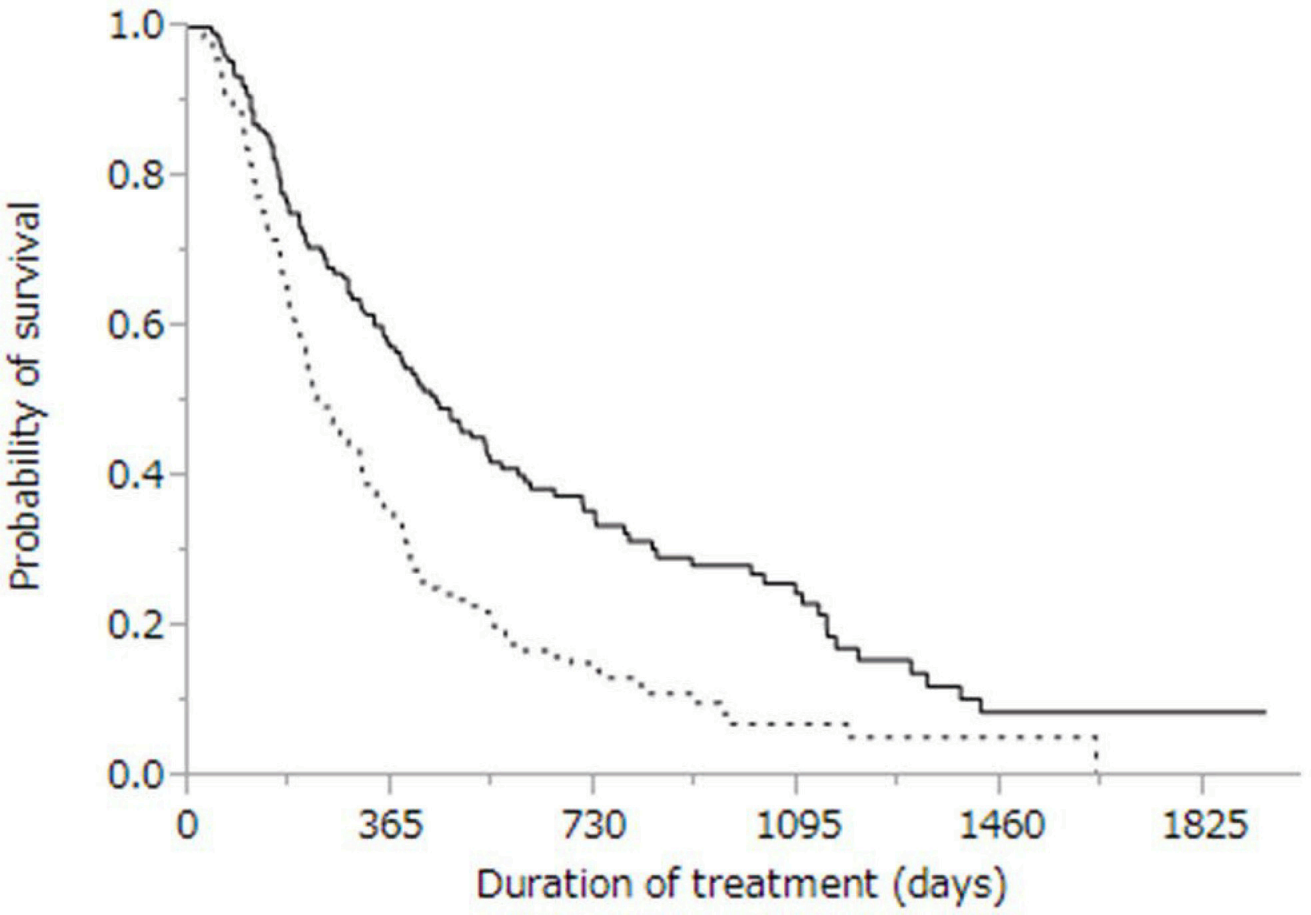
Kaplan-Meier analysis of overall survival of patients with disease control (solid line; n = 155) and with progressive disease (dotted line; n = 141) Median survival time was 14.9 months vs. 7.6 months, respectively (*p* < 0.0001).

The cumulative survival curves for all patients in Figures 1 and 2 yielded a median survival time of (MST) 10.6 months (range: 0.4–57.3 months), a 44% rate of one-year survival, and a median progression-free survival (PFS) time of 3.9 months (range: 0.1–34.3 months; Figure 2). Univariate analyses of overall survival identified eight baseline patient characteristics as prognostic indicators for overall survival: sex, Child-Pugh class, initial sorafenib dose, serum AFP level at baseline, serum AFP-L3 level at baseline, serum DCP level at baseline, treatment duration, and therapeutic effect (Table 3). Therapeutic effect was a significant risk factor in all patients. Figure 3 shows curves for cumulative survival of patients with disease control and PD, with respective MSTs of 14.9 and 7.6 months (*p* < 0.0001).

**Table 3:** Univariate analyses of overall survival in all patients

Variable	*p* value	HR (95% CI)
Age (≥72 years)	0.318	1.147 (0.876–1.502)
Gender (Male)	0.014	0.654 (0.476–0.916)
Etiology (HCV)	0.780	0.961 (0.731–1.272)
Child-Pugh class (B)	<0.001	2.136 (1.442–3.076)
Tumor stage (BCLC-C)	0.759	0.959 (0.732–1.258)
Initial sorafenib dose (800 mg)	0.005	0.690 (0.526–0.899)
Extrahepatic metastasis (With)	0.880	1.019 (0.795–1.310)
Macrovascular invasion (Presence)	0.168	1.216 (0.918-1.591)
AFP (≥100 ng/ml)	0.001	1.578 (1.204–2.072)
AFP-L3 (≥22.3 %)	0.002	1.654 (1.214-2.260)
DCP (≥738 mAU/ml)	<0.001	1.864 (1.398-2.443)
Duration of treatment (≥3.6 months)	<0.001	0.525 (0.396–0.692)
Therapeutic effect (PD)	<0.001	1.679 (1.268–2.225)

HR = hazard ratio; 95% CI = 95% confidence interval; HCV = hepatitis C virus; BCLC= Barcelona Clinic Liver Cancer; AFP = alpha-fetoprotein; DCP = des-gamma-carboxy prothrombin; PD = progressive disease

### Analysis of patients with PD after sorafenib

**Figure 4:**
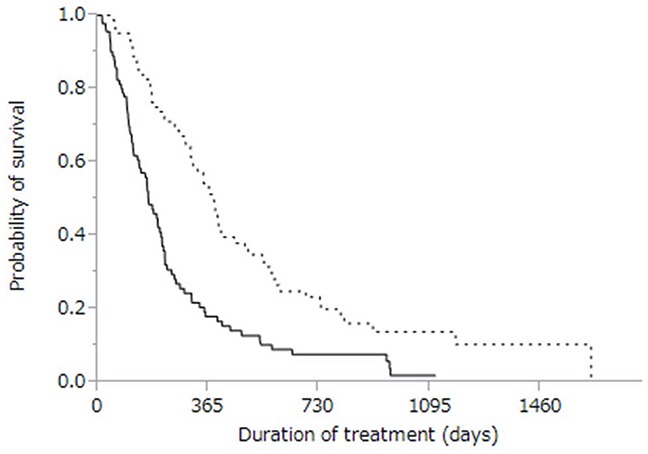
Kaplan-Meier analysis of overall survival in patients with progressive disease treated with sorafenib monotherapy (solid line; n = 58) and with therapies other than sorafenib (dotted line, n = 70) Median survival time was 6.1 months vs. 12.2 months, respectively (*p* < 0.0001).

Of 141 patients who developed PD after sorafenib treatment, 58 continued treatment with sorafenib monotherapy, and 13 received no treatment at all. Seventy patients received treatments other than sorafenib (Table 4). Cumulative survival curves of PD patients treated with either sorafenib monotherapy or alternative treatments are shown in Figure 4, with respective MSTs of 6.1 and 12.2 months (*p* < 0.0001). The MST for patients who received no treatment at all was 2.4 months.

**Table 4:** Additional treatments in advanced HCC patients with PD after sorafenib (n =141)

Additional treatment	n (%)
Sorafenib monotherapy	58 (41)
TACE	24 (17)
HAIC	21(15)
Systemic chemotherapy except for sorafenib	18 (13)
Radiation therapy	18 (13)
Hepatic resection	3 (2)
No treatment	13 (9)

HCC = Hepatocellular carcinoma; PD = progressive disease; TACE = transcatheter arterial chemoembolization; HAIC = hepatic arterial infusion chemotherapy

## DISCUSSION

Sorafenib is an oral multikinase inhibitor that has been recently used for molecularly targeted therapy of late-stage HCC patients [4]. Sorafenib treatment is well tolerated and has improved patient survival in two phase III clinical trials that were randomized and placebo-controlled [15, 16]. In our current study, we assessed prognostic indicators in advanced HCC patients, and evaluated alternative treatments in such patients who experienced PD after sorafenib. The MST (10.6 months) of sorafenib-treated patients was longer in our study (Figure 1) than in the Asia-Pacific study (6.5 months) [16] while being comparable to the MST observed for SHARP patients (10.7 months) [4, 15].

Using exploratory univariate analysis, we identified eight prognostic indicators of survival: sex, Child-Pugh class, initial sorafenib dose, serum AFP level at baseline, serum AFP-L3 level at baseline, serum DCP level at baseline, treatment duration, and therapeutic effect (Table 3). We demonstrated that PD after sorafenib was a risk factor that negatively affected survival (Figure 3). In line with other reports showing that early radiologic progression after sorafenib treatment is a sign of poor prognosis, patients in our cohort with early PD development also showed decreased survival [23, 28]. Although the purpose of administering sorafenib to advanced HCC patients is to maintain disease control over a long period of time, our data suggest that developing PD reduces the ability of sorafenib to provide survival benefits [29].

While previous clinical trials (SHARP and Asia–Pacific) showed that sorafenib can prolong the survival of patients with advanced HCC [15, 16], these studies made no recommendations as to whether sorafenib should be continued when PD first develops [14]. In our cohort, patients treated with alternatives to sorafenib survived longer than patients treated with sorafenib monotherapy (Figure 4) [30]. Therefore, our study suggests that sorafenib should be discontinued in favor of alternative treatments in advanced HCC patients with PD.

Hypoxia induced by some therapeutic approaches might promote tumor progression and metastasis [31]. It has been proposed that cancer cells can adapt to treatment and regenerate new tumors [32], and that carcinogen resistance may drive the accumulation of somatic mutations in oncogenes and tumor suppressors as an adaptive mechanism [33]. Therefore, drug combinations may be necessary to circumvent the problems of multiple mutations and drug resistance [34].

Currently, there are no effective second-line treatments for patients with HCC who have developed PD after being treated with sorafenib [23]. To improve prognosis for such patients, it is critical for clinicians to be able to predict tumor response to sorafenib treatment [21]. There are currently few biomarkers to predict the effects of sorafenib treatment; however, some genomic changes have been found to correlate with a favorable therapeutic response to sorafenib, and additional biomarkers might be identified in the next few years [21].

Our current study has several limitations. First, the alternative treatments in patients who developed PD after sorafenib treatment were selected at the discretion of the chief physician and were not randomized. This resulted in a selection bias for patients treated with sorafenib monotherapy and those administered alternative treatments. Second, some patients in the groups other than that administered sorafenib monotherapy received multiple treatments. Lastly, the size of the study cohort was relatively small. To confirm the superiority of alternative treatments (including the aforementioned combinatorial drugs) for abolishing sorafenib resistance in patients who develop PD after sorafenib treatment, prospective randomized studies with a larger number of subjects are required.

In conclusion, our results showed that PD after sorafenib treatment was a significantly negative prognostic indicator for patients with late-stage HCC. Those who developed PD after treatment with sorafenib and were administered alternative treatments had better survival potential compared to those who maintained treatment with sorafenib monotherapy. Therefore, alternative treatments for patients who develop PD after sorafenib treatment should be considered.
